# Serum hypomagnesemia is associated with febrile seizures in young children

**DOI:** 10.3934/Neuroscience.2022032

**Published:** 2022-12-26

**Authors:** Zakaria Ahmed Mohamed, Chunjiao Tang, Erick Thokerunga, Ali Omar Jimale, Jingyi Fan

**Affiliations:** 1 Department of Pediatrics, Zhongnan Hospital of Wuhan University, Wuhan 430071, China; 2 Department of Clinical Laboratory Medicine, Center for Gene Diagnosis, Zhongnan Hospital of Wuhan University, Wuhan 430071, China

**Keywords:** Febrile, seizure, magnesium, children, hypomagnesemia

## Abstract

**Background:**

Febrile seizures (FS) frequently manifest in children below 5 years of age. Although the exact etiology is still unknown, genetic predisposition, changes in neurotransmitter levels, and serum electrolyte imbalance are some of the known risk factors. This study examined the possible association between serum magnesium levels in children with FS compared to febrile children without seizures.

**Methods:**

A retrospective case-control study was conducted from February 2019 to January 2021, recruiting 230 age and gender-matched cases and controls (115 each). Extracted data were analyzed using SPSS using an independent student's t-test, Chi-square test, and Pearson's correlation analysis.

**Results:**

The mean serum magnesium levels were 0.93 ± 0.129 vs 0.97 ± 0.0961; p < 0.001, between cases and controls respectively. Similarly, hypomagnesemia (<0.85 mmol/L) was detected in 26.1% and 8.7% of the cases and controls, respectively; p < 0.001. A significant negative correlation was found between serum magnesium levels and the occurrence of febrile seizures; r = [−0.169], p < 0.05.

**Conclusion:**

Serum magnesium was significantly low in febrile children with seizures compared to those without, and hypomagnesemia was associated with the occurrence of febrile seizures. These results portray hypomagnesemia as a possible risk factor for febrile seizure, and so should be validated in future large cohort studies so that guidelines are set for proper management of these children.

## 1. Introduction

Febrile seizures (FS) are commonly manifested in children below 5 years of age [1]. They are defined as seizures occurring in children aged 6 to 60 months, with high-grade fever (>38 °C) and without central nervous system infection or metabolic disorders [2]. An estimated 2% to 5% of children in the USA and Western Europe suffer FS annually [3], compared to 8% to10% in Asia [4],[5]. The exact pathogenesis of FS remains poorly understood; however, genetic predisposition, changes in neurotransmitter levels, and serum electrolyte imbalance are some of the known risk factors [6]–[8].

Magnesium (Mg) is an abundant intracellular cation in the body. It is predominantly present in muscles, soft tissues, bones, and erythrocytes. Its major functions are to establish and maintain electrical potentials across cell membranes through the Na/K ATPases system, and inhibit voltage-gated calcium channels in the body. So, hypomagnesemia could trigger the release of calcium ions, which in turn induces nerve and muscle excitability [9]. In the brain, glutamate is the primary excitatory neurotransmitter, acting as an agonist of the N-Methyl-D-aspartate receptors (NMDA), while extracellular magnesium is known to inhibit these receptors. Therefore, hypomagnesemia can facilitate the excitation of NMDA receptors, allowing glutamate to depolarize the postsynaptic membrane and enhance epileptiform electrical activity [10].

According to a previous study, low serum magnesium level is associated with FS [11]. One study demonstrated that magnesium concentration in the cerebrospinal fluids (CSF) of epileptic children is significantly increased, potentially influenced by functional abnormalities in the cell membranes that may occur during epilepsy [12]. Although FS often have good prognoses, their occurrence is a worrisome predicament for parents of the affected children as about 2–8% eventually develop epilepsy in the future [13],[14]. A recent meta-analysis [15] summarized hypomagnesemia in children with FS, however, subsequent studies are still inconsistent [16]. In this study, we compared the levels of serum magnesium between children with febrile seizures and those who were febrile but had no seizures, to ascertain the hypothesized association between serum magnesium and febrile seizures.

## 2. Materials and methods

This case-control study was retrospectively conducted at the Department of Pediatrics, Zhongnan Hospital of Wuhan University, China, from February 2019 to January 2021. The study protocol was approved by the Zhongnan Hospital of Wuhan University Research Ethics Committee, and informed consent was sought from parents of all eligible children. Data from 115 cases (children with febrile seizures) and 115 age, and gender-matched controls (children with fever but no seizures) were retrieved from the hospital's electronic medical records and analyzed. Children included in the study were only those between the ages of 6 and 60 months, had FS, and had normal development. Similarly, age- and sex-matched control children with fever but no episodes of seizures were recruited. While, children with a history of congenital anomalies, infection of the central nervous system (CNS), metabolic disorder, and history of magnesium supplement receipt were excluded.

### 2.1. Data extraction and laboratory results

Extracted data included basic patient information, i.e., age of onset, sex, temperature, birth weight, gestational age, history of FS, family history of seizures, and serum magnesium levels. Laboratory results consisted of serum magnesium levels measured within 12 hours after the seizure. Abnormal results were considered to be those outside the reference range as set by our hospital laboratory; 0.85–1.15 mmol/L [17].

### 2.2. Data analysis

Statistical Package for the Social Sciences (SPSS) program version 22 was used for data analysis. Continuous variables were presented as mean ± SD and categorical variables as frequency & percentages. Independent student's t-tests were used to compare the means of continuous variables, while Pearson's Chi-square test was conducted for categorical variables. The correlation between hypomagnesemia and FS was determined by Pearson's correlation coefficient (r). Two-sided p-values of <0.05 was considered statistically significant.

## 3. Results

### 3.1. Demographic characteristics

In total, 230 age and sex-matched cases and controls were recruited, with mean age, 30.17 ± 14.5 vs 33.72 ± 13.31 months, p > 0.05 respectively. Mean birth weight was 3.23 ± 0.5136 vs 3.24 ± 0.513 kg, p > 0.05 for cases and controls respectively. Among the cases with preterm birth, 7.8% were premature, compared to only 1.7% of controls, p < 0.05). The mean temperature among the cases and the control group was 39.46 ± 0.66 vs 39.08 ± 0.57 °C, p > 0.05 respectively. In children with FS, fever was due to the respiratory and urinary tract infections in 99,1% and 0,9% of cases, respectively. All controls had a respiratory tract infection. Cases were more likely to present with a history of FS than controls, (36.5% vs 3.5%). Similarly, a family history of FS was more common in cases than controls, (33.9% vs. 0%). In terms of the type of FS, simple FS accounted for 94.7% of all febrile seizures, followed by complex febrile seizures at 5.3%. A detailed description is shown in Tables 1&2.

### 3.2. Hypomagnesemia in febrile seizures

Independent student's t-test for the standard mean difference of serum magnesium revealed that the mean serum magnesium level was significantly lower in the cases than the controls; 0.93 ± 0.129 vs 0.97 ± 0.0961 mmol / L; p < 0.01. Based on the laboratory interpretation of hypomagnesemia, (<0.85 mmol/L), 26.1% vs 8.7% of the cases and controls had hypomagnesemia respectively. Finally, Pearson's correlation analysis found that serum magnesium level was negatively correlated with FS (r = [−0.169], p < 0.05), suggesting that hypomagnesemia could have promoted the febrile seizures in these children. The detailed description is shown in Tables 3&4.

**Table 1.** Comparison of risk factors between 115 cases and 115 controls.

Parameters		Cases (n=115)	Controls (n=115)	P-value
**Age (months)**		30.17 ± 14.5	33.72 ± 13.31	1.719
**Gender**	Male	75 (65.2%)	75 (65.2%)	0.555
	Female	40 (34.8%)	40 (34.8%)	
**BW (kg)**		3.23 ± 0.5136	3.24 ± 0.513	0.844
**Gestational age**	Preterm	9 (7.8%)	2 (1.7%)	
	Term	106 (92.2%)	113 (98.3%)	0.03
**Temperature (°C)**		39.46 ± 0.66	39.08 ± 0.57	0.101
**Cause of fever**	Respiratory tract infection	114 (99.1%)	115 (100%)	
	Urinary tract infection	1 (0.9%)	0 (0.0%)	0.500
**History of FS**	Yes	42 (36.5%)	4 (3.5%)	
	No	73 (63.5%)	111 (96.5%)	0.000
**Family history of FS**	Yes	39 (33.9%)	115 (100%)	
	No	76 (66.1%)	0 (0.0%)	0.00

**Table 2.** Type of febrile seizures.

Type of FS	Frequency	Percentage
**Simple**	**109**	**94.7**
**Complex**	**6**	**5.3**
**Total**	**115**	**100**

**Table 3.** Comparison of serum magnesium levels between cases and controls.

Serum levels	Cases (n=115) Mean ± SD	Controls (n=115) Mean ± SD	P-value
**Magnesium**	0.93± 0.129	0.97 ± 0.0961	0.007

**Table 4.** Distribution of Hypomagnesemia in cases and controls.

Parameter		Cases freq. (%)	Controls freq. (%)	P-Value
Magnesium	Hypomagnesaemia	30 (26.1%)	10 (8.7%)	0.000
	Normal	85(73.9%)	105 (91.3%)	
Total		115 (100%)	115 (100%)	

Fishers exact test is applied; p value is significant, if <0.05.

## 4. Discussion

This study examined the relationship between serum magnesium and the occurrence of seizures in febrile children. By demographics, the cases and controls were comparable; the mean age was 30.17 ± 14.5 vs 33.72 ± 13.31 months in cases and controls respectively. However, this was higher than that observed by Namakin K et al, where the mean age of the children with FS was 24.1 ± 13.4 months [17]. Furthermore, in this study, approximately 65.2% of the cases were male and 34.8% were female, agreeing with Naseer et.al who also found 62% of febrile children with seizures were males versus 39% of females, indicating a male predisposition to FS [19].

In our study, the mean birth weights of cases and controls were comparable; (3.23 ± 0.51 vs 3.24 ± 0.51) kg, while the case group had more children with low gestational age than control group children (7.8% vs 1.7%). This result is consistent with that of another group [20]. We also found that the mean temperature at which FS occurred was 39.46 ± 0.66 degrees Celsius, consistent with report by Baaker et al. who found a mean temperature of 39.4 °C [21]. It has been documented that viral infections and temperatures above 39°C are associated with convulsive behavior in individuals [22]. The majority of our cases (99.1%) were due to respiratory tract infections, with cases having significant individual and family histories of febrile seizures than controls, 36.5% vs 3.5%, and 33.9% vs 0% respectively. This is also consistent with results from other published studies [23],[24]. By type of seizures, the vast majority of cases had simple FS (94.7%), compared to just 5.3% with complex FS, a result in agreement with Thakur et al., who found 78.8% of their cases had simple febrile seizures compared to 21.3% with complex FS.

The mean serum magnesium levels in our study were significantly lower in children with FSthan in febrile children without seizures and were negatively correlated with the occurrence of FS. Similar results were observed by Bharathi et al. [25] and Debroy et al. [26], while Baek et al. demonstrated that hypomagnesemia was an independent risk factor for FS (11). Talebian et al. simultaneously examined serum levels of magnesium and zinc in 60 children with FS, aged between 3 and 72 months, and found both grossly deficient. In this study magnesium and zinc supplements were recommended [27]. Similarly, Kannachamkandy et al simultaneously examined serum magnesium and copper in 35 children with febrile epilepsy and controls and discovered that while magnesium was significantly deficient, copper was rather higher in cases than in controls and that both levels were significantly associated with febrile seizures [28]. These studies suggest that there could be an association between magnesium and other trace elements in the serum of children with FS that need to be investigated. The muscle hyperexcitability seen in magnesium-deficient FS is possible because magnesium is required by enzymes that maintain cell membrane stability and nerve conduction, and so hypomagnesemia would lead to nerve and muscle excitability. Moreover, magnesium blocks the calcium channel in the NMDA receptor; consequently, it must be released for glutamatergic excitatory signaling to occur. Theoretically, low magnesium levels may enhance glutamatergic neurotransmission [29].

Despite the possible association between hypomagnesemia and febrile seizures observed in this study, the small sample size still limits the generalizability of the results. Future studies with large sample sizes are required to confirm these results.

## 5. Conclusion

In summary, this study revealed that serum magnesium was significantly low in febrile children with seizures compared to those without. Hypomagnesemia was also significantly associated with the occurrence of FS was more likely to occur in children with individual and family histories of FS. These results indicate that hypomagnesemia could be a risk factor for a febrile seizure. It is thus important that future large cohort studies look into this association to ensure that guidelines are set for the proper management of these children.
